# Supplementary material for: Are carbon emissions trading and green financial instruments synergistic? -Comprehensive quantitative research based on content analysis
Source: PLoS One. 2024 Mar 7;19(3):e0298601. doi: 10.1371/journal.pone.0298601 (PMC10919601; doi:10.1371/journal.pone.0298601)
Supplement: S1 Data — (ZIP) [file pone.0298601.s001.zip › Data/地级市Z_F工作报告.zip!/╜¡╬≈/╕╙╓▌╩╨/└┤╫╘╣1⁄2╓┌║┼í╛┬φ┐╦╩2╛▌═°í┐_2016.docx]

现在，我代表市人民政府向大会报告政府工作，请予审议，并请各位政协委员和列席会议的同志提出意见。

　　一、2015年和“十二五”工作回顾

　　过去一年，全市上下深入贯彻落实党的十八大、十八届三中、四中、五中全会和习近平总书记系列重要讲话精神，在市委的坚强领导下，全力以赴稳增长促发展，全市经济运行总体平稳，各项事业加快发展。主要经济指标保持中高速增长，实现地区生产总值1973.87亿元，增长9.6%，总量、增速均居全省第二；财政总收入353.32亿元，增长7.5%，其中一般公共预算收入245.51亿元，增长9%；一般公共预算支出614.97亿元，增长14.7%，总量保持全省第一；固定资产投资1892.21亿元，增长17.6%，增速连续四年全省第一；社会消费品零售总额705.21亿元，增长8.6%；城镇居民人均可支配收入25001元，增长9%；农村居民人均可支配收入7786元，增长12.1%，增速连续三年全省第一。

　　一是集中力量主攻工业，产业升级迈出新步伐。制定实施主攻工业三年推进计划、精准帮扶企业实施意见和考评方案，出台七个方面综合配套政策文件，建立市县两级领导挂点帮扶企业全覆盖机制，主攻工业势头良好。全市净增规模以上工业企业93户，总数达1188户；规模以上工业增加值753.26亿元，增长9.2%；家具产业成为继稀土钨之后第二个主营业务收入过千亿元的产业集群。新能源汽车整车生产取得突破性进展。国家（赣州）家具产品质量监督检验中心建成运行，青峰药业获批创建企业国家重点实验室。新增国家级博士后科研工作站3个。专利申请量和授权量均列全省第二，分别增长56.9%、101.2%。

　　粮食生产实现“十二连丰”。赣南脐橙以657.84亿元的品牌价值蝉联全国初级农产品类地理标志产品价值榜榜首，并荣获“2015最受消费者喜爱的中国农产品区域公用品牌”。“赣南茶油”获批国家地理标志保护产品。蔬菜、生猪等区域特色产业稳步发展。信丰县被认定为第三批国家级现代农业示范区。新增农民合作社1333家、家庭农场1327家，总数分别达5793和2137家。

　　引进中信银行、华融集团等金融机构5家，全市金融机构总数达166家。金融业实现增加值103.21亿元，增长20.9%。旅游接待总人数4801万人次，增长37.1%；旅游总收入395亿元，增长41.3%。瑞金共和国摇篮景区创建国家5A级旅游景区。赣州列入粤赣皖苏四省物流大通道节点城市。实施购房契税补贴、提高公积金贷款额度、推进棚改货币化安置等有效措施，房地产市场保持平稳健康发展。电子商务交易额达221.32亿元，增长101.1%，连续三年增长翻番。

　　二是深入推进改革开放，振兴发展增添新动力。商事制度改革“三证合一、一照一码”全面实施，企业信用监管警示系统上线运行。户籍管理制度改革扎实推进，中心城区实施户口“一元化”登记。农村土地承包经营权确权登记颁证工作基本完成。不动产统一登记改革进展顺利。金融改革创新取得突破。获批设立300亿元的赣南苏区振兴发展产业投资基金。新增“新三板”挂牌企业14家，上市挂牌企业总数达20家。“财园信贷通”等四个“信贷通”累计发放贷款108.7亿元。建立全口径财政预算编制体系，专项资金竞争性分配、稀土钨资源税从价计征改革成效明显。国有企业改革有序推进，组建混合所有制企业22家。开放型经济稳中有升。组织开展以“民企入赣、赣商回归”为主线的“13+5”系列招商活动，全市实际利用外资13.7亿美元，增长12.1%；引进省外5000万元以上项目资金600.28亿元，增长14.5%。实现进出口总额41.55亿美元，增长6.5%，其中出口总额33.92亿美元，增长6%。

　　三是着力抓好重大项目建设，城乡发展呈现新变化。127个市属重点工程项目累计完成投资603.3亿元，占年计划的118.5%。赣龙铁路复线正式通车，昌赣客专开工建设。寻乌至全南、南昌至宁都高速公路建成通车，大广、济广和寻乌至全南高速公路出省“断头路”全部打通。赣州黄金机场改扩建工程加快推进，瑞金支线机场选址获批。瑞金红都500千伏等22个输变电工程投入使用，华能瑞金电厂二期开工建设。290座一般小（2）型病险水库除险加固全面完成。黄金大桥、武陵大桥和南门口下穿隧道建成通车。中心城区创建国家园林城市通过复查，瑞金市被列为国家历史文化名城。27个全国重点镇、22个省百强中心镇、17个市级示范镇、606个新农村建设点和104个中心村建设扎实推进，8个乡村入选全国首批“美丽乡村”创建试点。

　　四是加大力度保障和改善民生，各项事业得到新发展。全市民生类支出350亿元，占财政总支出比重达56.8%。省、市安排的90件民生实事大部分超额完成全年任务。出台实施 “1+1+17”的精准扶贫政策体系，实行市县两级单位与1419个贫困村、市县乡三级干部与30.82万贫困户结对帮扶全覆盖，全年减少贫困人口34.82万人。完成城市棚户区改造1.01万户，货币化安置率达43.76%，其中中心城区货币化安置率达100%。新增中心城区商品蔬菜规模基地7400亩，新（改）建农贸市场5个。建成各类创业孵化基地和创业园区58个。新增城镇就业7.32万人，转移农村劳动力11.31万人。我市被确定为全国“救急难”综合试点单位。部省共建教育改革发展试验区建设取得实效。赣州职教园区建设进展顺利。全面推开县级公立医院综合改革和新农合大病保险，9种重大疾病免费救治惠及3.1万群众，市人民医院新院投入使用。公共文化服务标准化、均等化建设试点和客家文化（赣南）生态保护实验区建设扎实推进。市博物馆新馆开馆运行。成功举办第四届市运会。安全生产和信访形势保持稳定。国防动员、民兵预备役建设、人民防空、双拥共建和优抚安置等工作不断加强。

　　五是加强政府自身建设，行政效能得到新提升。深入开展“三严三实”专题教育，全面实施绩效管理考核，政府作风和效能建设明显加强。市县两级政府机构改革工作如期完成。机关事业单位工作人员工资标准调整到位。市本级行政审批项目由597项减为168项，缩减72%；平均办结时限由14.65个工作日缩短为7.4个，缩短近50%，非行政许可审批事项全面清理取消。将“三单一网”建设提升为“六单一网”，各项审批服务清单对外公布，政务服务网市县分厅上线运行。设立行政复议委员会，建立重大执法决定法制审核、行政执法信息公开和全过程记录制度，法治政府建设不断加强。自觉接受市人大法律监督和市政协民主监督，办理建议、提案533件，办结率、满意和基本满意率均达100%。严格监督执纪问责，政府廉政建设取得新成效。

　　各位代表，2015年是“十二五”圆满收官之年。“十二五”时期，是赣州发展迎来重大历史性机遇的时期，是赣南老区面貌发生巨大变化的时期，是赣南人民得实惠最多的时期，是赣州知名度和影响力提升最快的时期。全市经济社会发展取得显著成就，为“十三五”发展打下了坚实基础。

振兴发展取得阶段性重大成效。争取国务院出台《关于支持赣南等原中央苏区振兴发展的若干意见》，推动赣南苏区振兴发展上升为国家战略。国家层面支持赣南苏区的政策文件达106个，全面执行西部大开发政策基本到位，162项具体政策或标准已经落实，35项国家层面批复的试点示范事项加快推进，对口支援工作取得实效。龙南、瑞金经开区和赣州高新区升级为国家级园区，赣州综合保税区封关运行，赣州进境木材国检监管区和定南公路口岸作业区建成运营，赣州成为江西乃至全国拥有国家级平台最多的设区市之一。特别是改造农村危旧土坯房69.5万户，近300万农民告别透风漏雨的危旧土坯房；规划内农村人口安全饮水问题提前一年全部解决，278.4万人喝上安全卫生的饮用水；完成农村低电压治理41.39万户；新建改造农村公路1.37万公里，改造农村危桥556座，群众“出行难”问题得到有效缓解。《若干意见》提出的中期目标基本实现。

综合经济实力显著增强。主要经济指标增速明显高于全国、全省平均水平，地区生产总值接近2000亿元，年均增长10.9%。实现五个翻番：即财政总收入、一般公共预算收入、规模以上工业增加值、固定资产投资、出口总额实现翻番，分别是2010年的2.8、3.1、2.6、2.4和2.6倍；七个突破：即人均生产总值突破3000美元，财政总收入、一般公共预算支出分别突破300和600亿元，规模以上工业主营业务收入突破3000亿元，出口总额突破30亿美元，存、贷款余额分别突破3000和2000亿元。主要指标人均水平与全国全省差距缩小。产业结构更加优化，服务业占比明显提高。

　　基础支撑条件明显改善。新增铁路运营里程178公里，总里程达555公里，高铁建设和动车运营实现零的突破。新增高速公路通车里程473公里，总里程达1116公里，实现县县通高速。民航旅客年吞吐量突破90万人次。所有县城实现110千伏双电源供电，全市最大供电能力提升至340万千瓦，五年翻了一番。4G网络覆盖所有城区和乡镇以及80%以上行政村。南康撤市设区，中心城区建成区面积扩大至141平方公里，人口达141万。常住人口城镇化率达45.51%，年均提高1.59个百分点。

　　生态文明建设走在前列。国家森林城市、低碳试点城市、新能源示范城市和生态文明先行示范区建设深入推进。PM2.5监测、水土流失治理、重金属污染防治、农村垃圾专项治理取得阶段性成效，单位GDP能耗五年累计下降16.3%，主要污染物减排任务全面完成，县县建成城镇生活污水处理厂。全市空气质量稳定达到国家二级标准，集中式饮用水源地水质达标率保持100%，森林覆盖率稳定在76.2%以上，环境质量持续改善。城市生态环境竞争力进入全国前20强，成为全国首批创建生态文明典范城市。

　　民生福祉大幅提升。坚持每年办好一批民生实事，民生类支出占财政总支出比重连续四年超过55%。累计新建各类保障性住房18.96万套，实现人均住房面积15平方米以下困难家庭应保尽保。中心城区累计投放公交车607台，开通至瑞金、龙南、上犹的城际公交快线。覆盖城乡的社会保障体系和基层医疗卫生服务体系基本建成。累计新（改、扩）建校舍面积401万平方米，消除校舍危房211万平方米，建成乡镇及城区公办幼儿园322所，为70多万农村义务教育阶段学生免费提供营养餐。每千名老年人拥有养老床位数达到36张，高于全国平均水平5.2张。累计脱贫145万人，贫困发生率下降20.6个百分点。城乡居民人均可支配收入分别是“十一五”末的1.76和1.86倍。连续五届获“全国社会管理综合治理工作优秀市”，被授予第三届综治“长安杯”和全省首届综治“平安杯”，公众安全感指数保持全省前列。

　　各位代表，回顾过去一年及“十二五”发展历程，困难比预想的多，成效比预期的好，这主要得益于党中央、国务院的深切关怀，省委、省政府和市委的正确领导，国家部委和省直厅局的倾情支援，市人大和市政协的监督支持；得益于各级各部门强化使命担当，敢于创新破难，以抓铁有痕、踏石留印的精神推动工作落实；得益于全市人民齐心协力、团结拼搏、苦干实干，用辛勤劳动创造美好生活，谱写了赣南苏区振兴发展的壮丽篇章。在此，我谨代表市人民政府，向全市广大工人、农民、知识分子、干部和历任老领导、老同志，向各民主党派、工商联、无党派、人民团体和社会各界人士，向驻市人民解放军、武警官兵和公安干警，致以崇高的敬意！向中央和省对口支援部门，向所有关心、参与和支持赣州发展的同志们、朋友们、港澳同胞、台湾同胞、海外侨胞，表示衷心的感谢！

　　需要说明的是，随着我国经济发展进入新常态，全国、全省经济发展增速普遍放缓，宏观经济下行压力持续加大，对我市以资源型为主导的产业影响较大，加上政策性减税降费拉低财政收入增长、民间投资和房地产投资低位运行等因素，导致部分指标与年度目标增速还有差距。但面对严峻复杂的宏观形势，面对前所未有的困难和挑战，全市上下付出了非同寻常的艰辛努力，主要经济指标总量和增速仍然保持全省前列，实属来之不易。与此同时，我们也清醒地认识到，我市欠发达、后发展的基本市情没有根本改变，经济总量偏小、结构不优、后劲不足等问题仍然突出，转型升级任务艰巨；基础设施不够完善，城镇化水平较低，县域经济实力较弱；基本公共服务供给不足，贫困人口多，贫困程度深，面临小康提速和脱贫攻坚的双重压力；改革发展任务依然繁重，政府作风和效能与发展要求还有一定差距等等。对此，我们将在今后的工作中，采取更加有力措施，认真加以解决。

　　二、“十三五”发展展望

　　“十三五”时期是全面建成小康社会的决胜阶段。世界经济将在深度调整中曲折复苏，我国经济长期向好的基本面没有改变，省委“十三五”规划提出强化赣州在“苏区振兴”战略中的核心引领作用，推动赣州等原中央苏区整体跨越式发展、同步全面小康，这些积极因素，将为我市加快发展提供更加有利条件和更为广阔空间。特别是习近平总书记对江西工作提出了新的希望和“三个着力、四个坚持”的总体要求，为加快赣南苏区振兴发展注入了强大动力。随着中央打赢脱贫攻坚战和支持革命老区开发建设等战略决策深入实施，《若干意见》政策潜能日益释放，“六大攻坚战”全面推进，一大批重大基础设施和产业项目开工建设，赣州基础支撑能力、综合承载能力和内生发展动力将极大增强，只要我们保持定力、奋发有为，与全国全省同步全面小康的目标必将实现。

　　为科学制定全市“十三五”规划，描绘未来五年发展蓝图，市委深入开展一系列重大课题调研，邀请国家和省发改委专家现场指导，对接衔接国家和省“十三五”规划，广泛征求中央国家机关挂职干部、专家学者和社会各界意见建议，提出了《关于制定全市国民经济和社会发展第十三个五年规划的建议》，明确了“十三五”时期我市经济社会发展的主要目标：即地区生产总值、财政总收入、城乡居民人均可支配收入力争年均增长10%左右，工业三年翻番，脱贫摘帽三年完成。市政府据此编制了《赣州市国民经济和社会发展第十三个五年规划纲要（草案）》，提出了“实力提升、质量优化、生态宜居、人民富裕、社会和谐”5大类40项指标，其中预期性指标21项，约束性指标19项。确定这些目标，一是考虑到目前我市全面小康进程相对滞后，为实现与全国、全省同步全面建成小康社会，地区生产总值等指标必须保持高于全国、全省的增长速度；二是“十二五”期间，我市生产总值年均增长10.9%，“十三五”设置10%的目标，既与经济发展长期变化趋势相衔接，又反复测算了“十三五”时期我市潜在经济增长水平，通过努力能够实现；三是与省“十三五”规划相衔接，部分预期性指标和19项约束性指标的设置，与省里规划基本一致。

　　按照规划建议和《纲要》确定的宏伟目标，未来五年，将是赣州经济社会发展实现历史性跨越的五年。

　　——经济实力显著提升。到2020年，全市地区生产总值突破3000亿元，财政总收入突破500亿元，人均主要经济指标与全国、全省平均水平的差距明显缩小，赣州将成为全省最具潜力、支撑全省协调发展的重要增长板块。

　　——现代产业体系基本建立。规模以上工业主营业务收入突破8000亿元，形成一批千亿元产业集群和百亿元龙头企业，建成全国稀有金属产业基地、先进制造业基地和特色农产品深加工基地，四省边际区域性金融中心、物流中心、文化旅游中心和健康养老中心。

——老区面貌焕然一新。铁路建设及运营里程达到1300公里以上，赣州进入高铁时代，高速公路通车里程突破1600公里，25户以上自然村全部通水泥（油）路，形成覆盖城乡的比较完备的现代基础设施体系；中心城市人口突破200万，全市城镇化率达到53%以上，城乡一体化发展和基本公共服务均等化水平明显提升，赣州成为区域性综合交通枢纽、区域性现代化中心城市。

　　——振兴梦想如期实现。现行标准下农村贫困人口全面脱贫、贫困村全部退出、贫困县全部摘帽，覆盖城乡的社会保障体系比较完善，基本公共服务水平接近或达到全国平均水平，我国南方地区重要的生态屏障基本建成，人民生活水平和质量大幅提升，赣州与全国全省同步全面建成小康社会。

　　三、2016年工作安排

　　2016年是“十三五”开局之年。做好今年工作的总体要求是：认真学习贯彻党的十八大和十八届三中、四中、五中全会以及习近平总书记系列重要讲话精神，深入贯彻中央、省委经济工作会议和市委四届七次全会、全市经济工作会议暨扶贫开发工作会议部署，牢固树立创新、协调、绿色、开放、共享发展理念，认真落实省委“十六字”方针，坚持解放思想，内外兼修，北上南下，围绕打好“六大攻坚战”，抓项目、补短板、降成本、拓空间、强动力，为实现“十三五”经济社会发展目标任务开好局、起好步。

　　全市经济社会发展的主要目标是：生产总值增长9%；财政总收入和一般公共预算收入分别增长8%；规模以上工业增加值增长9.5%；固定资产投资增长17%左右；社会消费品零售总额增长10%；出口总额增长2%；实际利用外资增长10%；城乡居民人均可支配收入分别增长9%和11%；居民消费价格总水平涨幅控制在3%左右；城镇登记失业率控制在4.5%以内，节能减排完成省下达的计划任务。生产总值、财政收入等指标增速安排低于“十三五”规划目标，主要考虑当前宏观经济下行压力仍在加大，抓项目扩投资的潜能释放有个过程，“十三五”时期我市经济发展呈“前低后高”特征。今年的目标既与“十三五”规划相衔接，又体现实事求是，兼顾需要和可能。

　　为实现上述目标，今年我们将继续挖掘放大《若干意见》等国家扶持政策的叠加优势，提升对口支援实效，争取中央和省更大力度支持。按照市委每个攻坚战抓好十大项目或十件大事、建立“六个一”工作机制的部署，层层压实责任狠抓落实，确保“六大攻坚战”扎实推进，各项工作取得新成效。

　　（一）打好主攻工业攻坚战

　　着力帮扶企业发展。落实好精准帮扶企业的实施意见和企业帮扶全覆盖机制，发挥好工业经济运行监测分析平台、企业精准帮扶APP平台、中小微企业服务中心作用，因企施策、分类施策，支持龙头企业做大做强，小微企业发展壮大。开展降低企业运行成本行动，采取有力措施降低企业交易、人工、财务、物流成本，落实西部大开发税收优惠政策、“营改增”等结构性减税政策，降低企业税费、社保负担，降低用电价格。大力推广“财园信贷通”等模式，组建金盛源担保集团，完善地方政府倒贷机制，多措并举缓解企业资金困难。

　　培植壮大产业集群。坚持每个主导产业每年办好十件大事，抓好十个重点在建项目、十个重大招商项目，服务好十个重点企业，加快编制产业发展规划，出台配套扶持政策，整合资源要素推动产业集聚发展。以“稀金谷”建设为平台，以中国南方稀土集团等企业为龙头，建立“大数据”平台，整合勘探、分离、加工、研发、贸易等全产业链，推动稀土钨产业向高端新材料及应用领域升级。开工建设新能源汽车科技城，加快中航新能源汽车赣州产业基地、孚能科技动力电池及系统产业化等重大项目建设，引进战略资本以及整车生产和配套企业，做实新能源汽车“赣州制造”。依托质量检验中心等公共服务平台，推动南康家具标准化、品牌化发展，打造全国最大的实木家具加工和出口基地。积极推广应用新技术、新工艺、新装备、新材料，促进铜铝有色金属、电子信息、纺织服装、食品加工、建材、氟盐化工等产业转型升级。推动“互联网+”协同制造，促进新一代信息技术与制造业融合，培育壮大高端装备制造、生物制药、3D打印等新兴产业。引导各地用好赣南苏区振兴发展产业投资基金、产业发展基金、科技创业投资引导基金，设立市级工业发展引导基金，发挥财政资金杠杆效应，撬动吸引更多社会资本参与工业发展。

　　推动园区升级。结合各地产业发展基础条件，突出首位产业集聚发展，每个园区重点培植1-2个产业集群，走差异化、集约化、特色化发展路子。支持赣州经开区主攻新能源汽车及配套产业，赣州高新区围绕建设“稀金谷”重点发展稀土钨新材料产业，龙南、瑞金经开区着眼承接珠三角和海西经济区产业转移，建设赣粤（港）、赣闽（台）对接合作新区。支持园区扩区增容，大力开展标准厂房建设，完善基础设施和配套服务设施。进一步理顺园区管理体制，探索产城融合和空间整合，做大园区经济总量，争取在全省排位前移。

　　（二）打好精准扶贫攻坚战

　　健全扶贫机制。层层压实领导责任、主体责任、帮扶责任，制定时间表、路线图、任务书，确保全年脱贫25万人。完善贫困人口进退机制，做好扶贫对象认定、评估、退出工作，开展精准识别回头看、再复核，确保扶贫对象真实准确。完善单位定点扶贫、干部结对帮扶全覆盖机制，强化脱贫攻坚责任考核，形成强大合力打赢脱贫攻坚战。

　　创新扶贫方式。抓住产业脱贫这个核心，推动产业脱贫与搬迁脱贫、兜底保障脱贫互相融合、互为促进，通过选准一个产业、打造一个龙头、建立一套利益联结机制、扶持一笔资金、形成一套服务体系，破解产业脱贫难题。整合移民搬迁、农村危房改造、保障房建设、土地增减挂等资金，采取多种安置方式引导贫困户向县城、工业园区、中心镇、中心村梯度转移，并完善后续扶持政策确保稳得住、能致富。对丧失劳动力、没有经济来源的贫困群众，实行兜底保障。构筑新农合补偿、大病保险补偿、疾病医疗商业补充保险补偿、医疗救助四道保障线，坚决遏制“因病致贫、因病返贫”。

　　加强资金保障。建立健全财政扶贫资金持续增长机制，市本级今年安排脱贫攻坚专项资金3.95亿元，确保政府投入力度与脱贫攻坚任务相适应。拓宽资金来源渠道，整合专项扶贫资金、相关涉农资金和社会帮扶资金捆绑集中使用，加强资金监督管理，提高资金使用效益。市本级筹资10亿元作为风险缓释基金，力争撬动80亿元“扶贫信贷通”贷款帮助贫困群众发展产业。创新“油茶贷”、移民搬迁贷款、农房抵押贷款等金融产品，撬动银行及社会资金服务脱贫攻坚。

　　（三）打好新型城镇化攻坚战

　　加强城市规划建设管理。优化和完善“一区两群”城镇空间总体布局，加快以人为核心的新型城镇化进程，集中要素做大做强做美中心城区和县城，构建大中小城市和小城镇协调发展的城镇体系。高品位规划建设蓉江新城，完善老城区和章江新区功能，全面优化提升中心城区市政和生活配套设施水平。加快文明大道、迎宾大道、客家大道西延等城市快速路网建设，开工建设红旗大桥，建成客家大桥，构建城市各组团之间的快速通道。打通一批章江新区、河套老城区“断头路”，畅通城市交通“微循环”。开工建设地下综合管廊和一批公共停车场、小游园、小广场。有序推进历史文化街区修缮整治，稳步实施特色街道立面整治改造。继续推进城市管理工作重心下移，加大力度治理“脏、乱、堵”，争创全国文明城市。

统筹城乡发展。以示范镇、重点镇建设为突破口，以美丽乡村建设为抓手，突出公共服务和产业发展，着力打造一批人口集中、服务功能完善、产业兴旺、辐射带动力强的特色镇和农村新社区。抓好104个中心村和998个村点新农村建设。实施农村环境连片整治工程，完成300个村庄环境整治，继续推进农村生活垃圾专项治理，打造特色鲜明的农村人居环境改善示范样板。

　　壮大县域经济。围绕促进县域经济发展放权、让利，在基础设施、重大项目建设以及融资、科技人才引进等方面予以更多倾斜，服务指导各县（市、区）打好“六大攻坚战”。出台县域经济发展规划和意见，完善县域经济发展考评体系，支持各县（市、区）立足县情，选好首位产业，培植发展一批龙头企业，集聚发展一批配套产业，做强工业、夯实基础、改善民生，补齐县域经济短板。

　　（四）打好现代农业攻坚战

　　发展优势特色产业。坚持“藏粮于地、藏粮于技”，巩固提升粮食产能。打好柑橘黄龙病防控“歼灭战”，加强赣南脐橙标准化体系和质量追溯体系建设。推广生态化、机械化果园建设，打造100个标准果园。稳定规模、调优结构、提高效益，推动脐橙产业发展升级。新造、改造高产油茶林25万亩，加快油茶“三个中心、三个基地”建设，做强赣南油茶品牌。推进蔬菜产业发展，力争中心城区新增规模蔬菜基地8000亩。大力发展花卉苗木、毛竹、茶叶、生猪、白莲、刺葡萄等区域特色产业。抓好国家、省级现代农业示范区和“百县百园”现代农业示范园区建设，引领现代农业发展，促进农民增收。

　　构建新型农业经营体系。推动农业生产与农产品加工、流通、服务有机结合，加快农产品批发交易市场和仓储、物流设施建设，促进农业接二连三。健全完善基层农技推广体系，扎实开展新型职业农民培育。推进适度规模经营，培育壮大龙头企业、种养大户、农民专业合作社、家庭农场等新型农业经营主体。建立健全从生产到消费各环节可控的农产品质量安全保障体系，确保不发生重大农产品质量安全事件和区域性重大动植物疫情。

　　深化农业农村改革。全面完成农村土地承包经营权确权登记颁证工作，建设农村产权流转交易平台；稳步推进农村宅基地制度改革，开展房地一体的农村集体建设用地和宅基地使用权确权登记发证工作。推进农村集体资产股份权能改革试点，深化小型水利工程管理体制改革、供销合作社综合改革，增强农村发展活力。

　　（五）打好现代服务业攻坚战

　　着力壮大金融业。围绕建设四省边际区域性金融中心和全省次金融中心，加快金融改革创新。组建赣州金融控股集团，争取设立地方法人寿险公司、公募基金管理公司，实现各县（市）农商行全部挂牌开业。大力发展科技金融、消费金融、融资租赁等业态，开展民间融资登记服务、商业保理等创新试点，探索保险资金在赣州创新运用。做大做强赣南金融资产交易中心，支持企业上市和发债，培育资本市场的“赣南板块”。坚决打击非法金融活动，维护金融稳定。

　　打响赣南文化旅游品牌。按照“一核三区”旅游产业空间布局，加快旅游资源整合开发。启动建设大型文化旅游主题公园，整合历史文化街区及赣州水系沿岸景观资源，打造“三江六岸”水上观光旅游景观带，推动宋城文化旅游核心区建设取得突破。抓好国家旅游扶贫试验区建设，实施旅游扶贫“十百千”工程，发展特色乡村旅游。

　　促进物流、电商等产业集聚发展。围绕建设区域性物流枢纽和重要节点城市，积极发展第三方物流和多式联运，大力发展农村物流，培育一批物流企业，提升物流信息化水平。扎实推进国家电子商务示范城市创建，抓好国家电子商务进农村综合示范县建设，打造跨境电商和稀有金属、家具、赣南脐橙四大电商交易平台。加强房地产市场监测预警，落实鼓励农民购房新政，推进棚改货币化安置，打好土地、财税、金融、公积金等政策组合拳，促进房地产业平稳健康发展。加快发展文化创意、体育休闲、健康养生、养老服务等新兴服务业。

　　（六）打好基础设施建设攻坚战

　　加快形成立体交通网络。围绕建设重要的区域性综合交通枢纽，加快昌赣客专建设，争取开工赣深客专、吉永泉铁路，做好渝长厦（井冈山至赣州段）、鹰瑞梅铁路等项目前期工作。建成兴国至赣县、宁都至安远、安远至定南（含定南联络线）高速公路，开工建设广昌至吉安高速公路宁都段。抓紧赣州黄金机场改扩建工程。加快国省道干线公路升级改造，推进 “三南”公路建设，提升农村公路通行能力。

　　增强能源保障能力。加快华能瑞金电厂二期工程建设，做好信丰、宁都煤电项目和赣县抽水蓄能电站前期工作。抓好30项110千伏及以上输变电工程建设，实现220千伏变电站县县全覆盖。推进省天然气管网二期工程、城市天然气管网工程建设。

　　完善水利基础设施。进一步完善以防洪、水资源供给、水生态保护为核心的水利综合支撑保障体系。实施五河治理防洪工程和县城防洪工程。加快灌区续建配套与节水改造，完成17个小农水重点县（项目县）建设。

　　提升信息化建设水平。推进“宽带中国”示范城市和“无线城市”、“光网城市”、“宽带乡村”建设。抓好“数字赣州”地理空间框架、智能交通指挥系统、小微企业信用信息服务平台等项目建设，规划建设大数据、云计算中心。

　　（七）大力实施创新驱动

　　加强企业创新能力建设。鼓励企业加大研发投入，建立研发机构，联合高校和科研院所组建科技创新联盟，开展产学研联合攻关。加快高新技术企业培育发展，力争高新技术企业增长20%以上。实施知识产权入园强企“十百千万”工程，开展知识产权质押融资试点，力争企业专利申请量、授权量均增长25％以上。

　　推动重点产业创新。加快新能源汽车工程研究中心建设，争取国家离子型稀土资源高效开发利用工程技术研究中心、国家脐橙工程技术研究中心通过验收。着眼稀土钨产业发展最前沿最高端，找准制约产业发展的关键环节，充分发挥国家级科技创新平台作用，整合国内外力量开展协同攻关，在新材料及应用领域突破一批核心技术，推动产业链延伸拓展。加快发展高技术服务业。搭建线上、线下科技成果转移转化服务平台，推进科技金融深度融合，集中资源扶持一批优秀创新成果转化为现实生产力。

　　鼓励大众创业万众创新。放宽政策、放开市场、放活主体，加快构建有利于创业创新的政策环境、制度环境和公共服务体系。实施“优秀高层次专业技术人才引进计划”和“赣州籍人才回归计划”，加强高技能人才、创业创新人才引进培养。建设一批“双创孵化器”，培育一批“双创示范基地”和“双创服务”平台，大力发展创业咖啡、创新工场、梦想小镇等新型众创空间。

　　（八）深化改革开放

　　深化重点领域改革。围绕“三去一降一补”推进供给侧结构性改革，提高供给体系质量和效率，培育发展新动能。进一步深化商事制度改革，简化和完善企业、个体工商户注销流程，探索企业登记全程电子化。继续抓好不动产统一登记改革，建设不动产登记信息管理基础平台。深化国资国企改革，推进国有资本运营公司试点，实现市属国有经营性资产监管全覆盖。加快国有企业市场化整合重组，引进战略投资者参与我市国有企业混合所有制改革。加快财税体制改革，进一步完善政府预算体系，建立健全政府性债务管理制度、预警机制，推进国税、地税服务深度融合、执法适度整合、信息高度聚合。深化投融资体制改革，大力推行政府和社会资本合作（PPP）等融资模式，促进民间投资和民营经济发展。有序推进水、电及自然资源等价格改革，完善主要由市场决定价格的机制。

着力招大引强。以招商引资大数据中心建设为抓手，进一步创新招商方式。瞄准世界500强、央企和大型民企等目标企业，大力开展专业招商、以商招商。围绕主导产业强链、延链、补链，组建专门招商队伍开展精准招商。大力实施“赣商回归、赣人兴赣”工程，鼓励和吸引在外赣商回乡创业兴业。建立重大招商项目市县联审、异地落户、利益分享等机制，狠抓招商项目落地投产。

　　提升开放合作水平。全面对接参与“一带一路”和长江经济带建设，主动对接广东、福建自贸区，探索与广东、福建跨区域合作共建产业园区。加快赣南承接产业转移示范区、瑞兴于经济振兴试验区等重大平台建设，提升赣州综合保税区、赣州进境木材国检监管区、定南公路口岸作业区运营水平，实施赣州铁路口岸作业区改造，建设龙南陆路口岸作业区。申报建设“中国服务外包示范城市”，争取开通赣州至深圳铁海联运快速班列。加快构建关检合作“三个一”、国际贸易“单一窗口”新型通关模式，培育外贸竞争新优势，做大外贸总量，推进外贸优进优出。

　　（九）建设生态文明先行示范区

　　实施绿色工程。按照生态文明先行示范区建设总体规划，继续推进“净空、净水、净土”行动。加强空气环境质量监测预警，推进“煤改电”“煤改气”工程和重点行业脱硫、脱硝、除尘改造，关停搬迁污染企业，淘汰黄标车。加大县城、工业园、中心镇污水处理设施及管网建设改造力度，提升污水处理能力和效果。加强重点区域水土流失综合治理、重金属污染防治和土壤污染源头综合整治。完成造林50万亩，改造低质低效林40万亩，争创国家森林城市。

　　发展绿色产业。将“生态+”理念融入产业发展全过程。大力发展无公害农产品、林下经济产品、绿色食品和地理标志农产品。推进全国餐厨废弃物资源化利用和无害化处理试点城市建设。实施好赣州经开区“国家园区循环化改造试点”。加快绿色矿业发展示范区建设。以节能、节水、节材、节地、资源综合利用为重点，实行能源和水资源消耗、建设用地等总量和强度双向控制，节约高效利用资源。

　　完善绿色制度。落实生态保护红线制度，完善生态红线内管制措施，加强耕地和基本农田保护。推动东江源、赣江源生态补偿机制试点，争取更多县（市）列入国家重点生态功能区。健全区域与流域相结合的“河长制”组织体系，全面开展“河长制”试点，抓好河湖保护管理突出问题整治，逐步实现河畅、水清、岸绿、景美。加强崇义、安远生态文明先行示范县建设，争创第二批全省生态文明先行示范市和县（区）。健全生态文明考核和责任追究制度，着力培育绿色文化，打造美丽中国的“赣州样板”。

　　（十）着力补齐民生短板

　　大力实施民生工程。整合300亿元以上资金，办好40件民生实事，并完成省下达的50件民生实事。完成棚户区改造2.92万户。中心城区新建社区蔬菜便利店60家，新（改、扩）建老旧农贸市场10个。新建改造农村公路3500公里，改造危桥100座。落实高校毕业生就业促进和创业引领计划，扩大就业总量，提高就业质量。完善多层次社会保障体系，提高城乡低保、农村五保等保障水平。新（改、扩）建福利院、敬老院、光荣院60所，发展居家养老、社区养老、医养结合等多种养老模式。深入推进“救急难”综合试点工作。加强综合防灾减灾能力建设。

　　协调发展各项社会事业。加快编制实施教育、卫生等社会事业发展专项规划，提升基本公共服务质量和水平。实施第二期学前教育三年行动计划。推进义务教育学校标准化建设，优化教育资源配置，缓解城区大班额问题。加快赣州职教园区及赣南职业技术学院建设。深化医药卫生体制改革，整合城镇居民医保和新农合，建立统一的城乡居民医保制度。实施城乡居民大病保险，推开医疗责任保险。启动市妇幼保健院新院建设，建成市食品药品检验检测中心。实施一对夫妇可生育两个孩子政策，促进人口均衡发展。深化国家公共文化服务体系示范区创建，推进“两馆一站”免费开放、广电“户户通”、农村文化“三项活动”等重大文化惠民工程，开工建设市文化艺术中心。实施全民健身计划，大力发展体育事业。加强国防动员和后备力量建设，深入开展双拥共建，巩固和发展军民团结。

　　营造和谐稳定的社会环境。加强公共法律服务体系建设，启动“七五”普法工作，建设法治赣州。加快立体化社会治安防控体系建设，完善社会矛盾纠纷多元化解机制。改造提升一批老旧社区、小区，创新城乡社区治理。推进诚信体系和信用平台建设，建立健全信用等级评估和失信联合惩戒机制。落实网上信访、逐级走访、诉访分离、法定途径分类处理信访投诉请求等信访改革政策，打造阳光信访、法治信访。扎实抓好安全生产，严防重特大安全事故发生，增强人民群众安全感。

　　四、加强政府自身建设

　　打好“六大攻坚战”，实现“十三五”良好开局，需要政府更加重视自身建设，不断提升能力水平。我们将坚持解放思想，创新方式方法，强化责任担当，狠抓落实执行，确保全年目标任务落到实处、取得实效。

　　着力打造法治政府。坚持将法治思维和法治方式贯穿政府工作全过程，严格按照法定权限和程序行使权力、履行职责。用好用足地方立法权，加强重点领域政府立法。完善重大决策合法性审查、社会稳定风险评估和责任追究机制，坚持依法科学民主决策。推进综合行政执法改革，促进公正文明执法。自觉接受人大法律监督、政协民主监督和社会舆论监督，办好各类建议和提案。

　　着力打造高效政府。持续推进简政放权、放管结合、优化服务，抓好事中事后监管，加强行政审批目录管理，完善行政权责清单动态调整机制，严禁清单之外擅自设权、行权。加大政务公开力度，畅通民意诉求表达渠道，及时回应群众关切。建设市县乡村四级政务服务平台，将实体政务大厅向网上办事大厅延伸，提升政府信息化建设和网上服务水平。加大绩效考核、督查督办和行政问责力度，优化政务环境，提升行政效能。

　　着力打造廉洁政府。驰而不息落实中央八项规定精神，促进作风建设常态化、长效化。严格落实党风廉政建设责任制，加大惩治和预防腐败力度，维护风清气正的政治生态。强化政府内部权力监督制约，在权力集中部门和重点岗位探索分事行权、分岗设权、分级授权。加强行政监察和审计监督，抓好民生和重点工程建设等领域监管。全面落实公务用车改革，控制和压缩“三公”经费，坚持勤俭办一切事业，努力建设节约型机关。

　　各位代表，宏伟蓝图已经绘就，奋斗号角已经吹响。让我们更加紧密地团结在以习近平同志为总书记的党中央周围，在市委的坚强领导下，向改革开放要动力，向创新创业要活力，向特色优势要竞争力，加快赣南苏区振兴发展，奋力夺取全面建成小康社会决胜阶段新胜利！
